# Effects of sleep habits on acute myocardial infarction risk and severity of coronary artery disease in Chinese population

**DOI:** 10.1186/s12872-021-02251-8

**Published:** 2021-10-07

**Authors:** Xiaoqing Lian, Jie Gu, Sibo Wang, Jianjun Yan, Xiaowen Chen, Mingwei Wang, Yuqing Zhang, Liansheng Wang

**Affiliations:** 1 Department of Cardiology, The First Affiliated Hospital of Nanjing Medical University, 300 Guangzhou Road, Nanjing, 210029 Jiangsu Province China; 2 Department of Cardiology, The Affiliated Jiangning Hospital of Nanjing Medical University, Nanjing, 211100 China; 3 Department of Cardiology, The Affiliated Hospital of Hangzhou Normal University, Hangzhou, 310015 China

**Keywords:** Sleep habits, Acute myocardial infarction, Coronary artery disease, Gensini score, Late sleeping

## Abstract

**Background:**

Growing evidence indicates that poor sleep harms health. Early to bed and early to rise is considered as a healthy lifestyle in Chinese population. The current study aimed to examine the effects of sleep habits on acute myocardial infarction (AMI) risk and severity of coronary artery disease (CAD) in Chinese population from two centers.

**Methods:**

A total of 873 patients including 314 AMI cases and 559 controls were recruited from the inpatient cardiology department of the Affiliated Jiangning Hospital and the First Affiliated Hospital of Nanjing Medical University. 559 controls included 395 CAD cases and 164 non-CAD cases. We used a 17-item sleep factors questionnaire (SFQ) to evaluate sleep habits comprehensively by face-to-face interview. The severity of CAD was assessed by Gensini score in AMI and CAD groups. The effects of sleep factors on AMI risk and Gensini score were examined by unconditional logistic regression.

**Results:**

After mutually adjustment for other sleep factors and demographic characteristics, the timing of sleep (24:00 and after) and morning waking (after 7:00) and sleep duration (< 6 h) were associated with increased risk of AMI (OR = 4.005, *P* < 0.001, OR = 2.544, *P* = 0.011 and OR = 2.968, *P* < 0.001, respectively). Lower level of light exposure at night was correlated with reduced risk of AMI (OR = 0.243, *P* = 0.009). In subgroup analysis by age, both late sleep timing and short sleep duration were associated with increased risk of AMI regardless of age. In subjects with age ≤ 65 years, daytime napping was related to reduced risk of AMI (OR = 0.645, *P* = 0.046). In subjects with age > 65 years, the frequency of night-time waking (3 times) was associated with increased risk of AMI (OR = 3.467, *P* = 0.035). Short sleep duration was correlated with increased risk of high Gensini score (OR = 2.374, *P* < 0.001).

**Conclusion:**

Sleep insufficiency is an important risk factor both for AMI risk and CAD severity. Late sleeping is also associated with increased risk of AMI. In young and middle-aged people, regular naps may have a protective effect.

**Graphic abstract:**

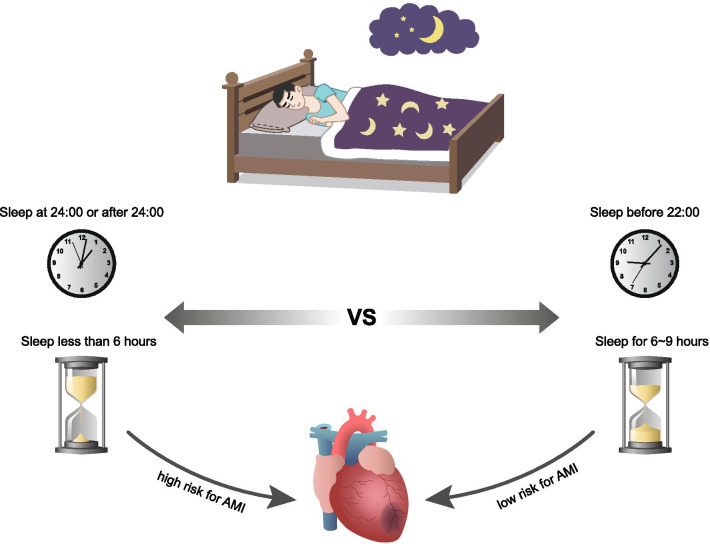

**Supplementary Information:**

The online version contains supplementary material available at 10.1186/s12872-021-02251-8.

## Introduction

It has been reported that sleeping between 7 and 9 h per night is the best choice for adults [1]. However, sleep insufficiency caused by stressful work and addiction to cell phone has become a common phenomenon in real world life. Growing evidence indicates that poor sleep harms health, either physical or psychological.

Short sleep and poor sleep quality increase the risk of cardiovascular disease mainly through influencing glucose metabolism and blood pressure and leading to insulin resistance and metabolic syndrome [2]. A longitudinal study proved that sleep for 6 h or less per night lost less weight and increased the risk of type 2 diabetes mellitus [3]. Unsurprisingly, consistent results showed that short duration of sleep increased the incidences of obesity and metabolic syndrome [4, 5]. Improving sleep quality was helpful to weight control [6]. Compared with individuals with 7 h of sleep per night, those with 6 h or even shorter had a higher risk of hypertension [7]. The relationship between sleep duration and coronary artery disease (CAD) displays a U-shaped curve, which means that short or long sleep is associated with a high risk of CAD. The association between long sleep and CAD risk has been verified in a meta-analysis [8].

Moreover, much evidence supports that CAD is closely correlated with sleep-related factors, such as sleep quality, siesta, insomnia, shift work and so on [9–12]. Gensini score is calculated based on the location and severity of stenosis in coronary lesions [13]. Patients of higher Gensini score are usually accompanied with moderate to severe obstructive sleep apnea [14], suggesting that a positive correlation existed between Gensini score and the severity of sleep apnea [15].

However, the effect of timing of sleep on cardiovascular disease and Gensini score has received scant attention. Early to bed and early to rise is considered as a healthy lifestyle in Chinese population, especially in the elderly. In the same night sleep duration, the elderly Chinese go to bed and get up approximately 1 h earlier than the elderly Europeans [16]. An interesting finding is that chronotype is associated with the onset time of acute myocardial infarction (AMI) [17], indicating that AMI incidence differs in different sleep–wake habits schedules.

Therefore, whether the traditional early sleep–wake habits could reduce the risk of AMI and severity of CAD is worth discussing. We designed this study to explore the influences of sleep habits on AMI risk and Gensini score in Chinese population from two centers.

## Methods

### Subjects

**Fig. 1:**
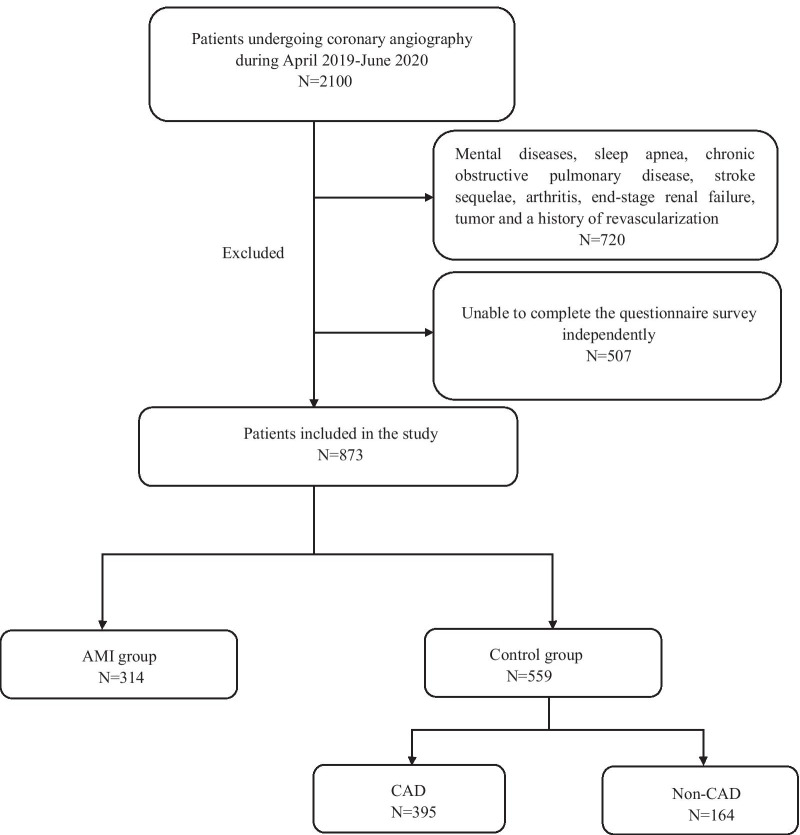
A flow chart of the inclusion and exclusion of patients

From April 2019 to June 2020, a total of 873 patients were consecutively recruited from the inpatient cardiology department of the Affiliated Jiangning Hospital and the First Affiliated Hospital of Nanjing Medical University. Patients hospitalized and diagnosed as non-AMI during the same time period were matched. Of them, 314 cases were AMI and 559 cases were controls (395 CAD and 164 non-CAD). All patients underwent coronary angiography and CAD was defined as at least one main coronary artery with > 50% narrowing of luminal diameter. Patients were diagnosed as MI when a cardiac biomarker (preferably cardiac troponin) rose or fell at least one value in its 99th percentile upper reference limit and at least one of the following criteria was met, including ischemic symptoms, electrocardiogram (ECG) changes of new ischemia, pathologic Q waves in the ECG, imaging evidence of new loss of viable myocardium or new regional wall motion abnormality, identification of an intracoronary thrombus by angiography or autopsy [18]. Patients were excluded if they had mental diseases [19], sleep apnea [20], chronic obstructive pulmonary disease [21], stroke sequelae [22], arthritis, end-stage renal failure, tumor and a history of revascularization. The definitions of above-mentioned exclusion criteria are provided in the Additional file 1. Figure 1 presents the details of the inclusions and exclusions. The Ethical Committee of the Affiliated Jiangning Hospital and the First Affiliated Hospital of Nanjing Medical University approved the study. All participants provided written informed consent as there were no interventions.

### Study design

We conducted a retrospective study in two centers. When patients were in stable condition during hospitalization, one trained interviewer administered a questionnaire survey by a face-to-face interview to evaluate sleep habits comprehensively in the last year before enrollment. And the participants were re-interviewed by another trained physician on a different day to ensure data valid and reliable. Information on demographic characteristics, hypertension, diabetes and dyslipidemia history, drug use, smoking, drinking, dietary habits and exercise were collected. We used Gensini score to evaluate CAD severity in AMI and CAD groups (detailed in Additional file 2) [23]. The result of coronary angiography was reported by two experienced interventional cardiologists. If the viewpoints of the two cardiologists were inconsistent, a third expert was consulted. Body height and weight were measured for all patients and body mass index (BMI) was calculated as weight (kg) divided by the square of height (m^2^).

### Sleep study

We employed a 17-item sleep factors questionnaire (SFQ) which had been proven to be reliable and valid previously to obtain information on sleep habits [24]. SFQ included sleep quality, sleep duration at night, timing of sleep and waking up, insomnia and night-time waking frequency, sleep medication use, night work, daytime napping, light at night (LAN) exposure and sleep noise (see Additional file 1 for definitions of these variables). Sleep quality was categorized as very poor, fairly poor, fairly good and very good by self-evaluation. LAN exposure was categorized into four levels: (1) they could read comfortably; (2) they could barely read; (3) they could see only the hazy outline of the bedroom; and (4) participants wore a mask to keep out light, or they could not see their hand in front of their face [24]. Sleep duration was divided into < 6 h, 6-9 h, > 9 h. The habitual timing of sleep was classified into before 22:00, 22:00 to 23:00, 23:00 to 24:00, and 24:00 and after. The timing of morning waking was classified into before 6:00, 6:00 to 7:00, and after 7:00. Napping at least 5 days per week was defined as regular daytime napping [25].

### Statistical analysis

Non-normally distributed continuous variables were expressed as median (interquartile range) and Mann–Whitney U-test was used to compare medians. Categorical variables were expressed as number (proportion) and the Chi-square test was used to test the distribution. When we analyzed the association between Gensini score and sleep factors, patients with no or mild coronary artery stenosis in non-CAD group were eliminated. Gensini score was divided into two groups by median value. The associations between sleep factors and AMI risk and Gensini score were examined by unconditional logistic regression. Every demographic variable was tested by univariate logistic regression and the variables with *P* < 0.1 and risk factors of AMI were adjusted in multivariate logistic regression. Besides adjustment for demographic variables, sleep factors were adjusted for each other. Spearman correlation was used to access the relationships among sleep factors and the sleep factors without relation for each other were included in logistic analysis. Age 65 was the cutoff point for subgroup analysis because the middle-aged and elderly people are divided by the age. The adjusted odds ratios (ORs) and 95% confidence intervals (CIs) were calculated. The difference was considered as significant if *P* < 0.05. All analyses were carried out using SPSS software (version 20.0).

We applied professional statistics software (PASS 15.0.1 Sample Size Software) to calculate the sample size. Considering the actual situation, the ratio of AMI to control group was set as 1:2. We expected a sample size large enough to detect an odds ratio of 2.0 with 90% power at the 0.05 significance level with a two-sided test. According to the 27% incidence of sleep disorders (WHO report), PASS software suggested that the sample size should be greater than 436 cases.

## Results

### Comparison in demographic characteristics between AMI and control groups

We enrolled 873 patients in total, with 314 AMI patients and 559 controls (395 CAD and 164 non-CAD). The characteristics and comparisons between AMI and controls are presented in Table 1. The comparisons between AMI and CAD group and non-CAD group are shown in Additional file 3. The median age was lower and the proportion of men were significantly higher in the AMI group than in the control and CAD groups (all *P* < 0.05). The education level, hypertension, use of ACEI or ARB, smoking, drinking, diet and regular exercise of the AMI group also significantly differed from the control group (all *P* < 0.05).

**Table 1:** Demographic characteristics of patients with AMI and controls

Variables	AMI group (n = 314)	Control group (n = 559)	*P* value
Age (years)	59 (51, 68)	63 (55, 69)	0.010
Men (n, %)	250 (79.6%)	361 (64.6%)	< 0.001
BMI (kg/m^2^)	25.1 (22.8, 27.0)	24.6 (22.8, 27.1)	0.509
Residence in cities (n, %)	239 (76.1%)	393 (70.3%)	0.070
**Education level (n, %)**			
Illiterate	40 (12.7%)	116 (20.8%)	0.001
Elementary school	58 (18.4%)	138 (24.7%)	
Junior high school	101 (32.2%)	140 (25.0%)	
Senior high school and technical secondary school	63 (20.1%)	98 (17.5%)	
Junior college or above	52 (16.6%)	67 (12.0%)	
Diabetes (n, %)	54 (17.2%)	119 (21.3%)	0.157
Hypertension (n, %)	165 (52.5%)	340 (60.8%)	0.019
Dyslipidemia (n, %)	20 (6.4%)	28 (5.0%)	0.440
Family history of CAD (n, %)	15 (4.8%)	16 (2.9%)	0.181
Statins (n, %)	11 (3.5%)	33 (5.9%)	0.147
Antiplatelet agents (n, %)	21 (6.7%)	51 (9.1%)	0.249
β-blockers (n, %)	13 (4.1%)	42 (7.5%)	0.059
ACEI or ARB (n, %)	54 (17.2%)	154 (27.5%)	0.001
**Smoking (n, %)**			
Never	102 (32.5%)	276 (49.4%)	< 0.001
Former	30 (9.6%)	82 (14.7%)	
< 20 cigarettes/day	46 (14.6%)	66 (11.8%)	
≥ 20 cigarettes/day	136 (43.3%)	135 (24.2%)	
**Drinking (n, %)**			
No	154 (49.0%)	349 (62.4%)	0.002
1 to 3 times a month	65 (20.7%)	88 (15.7%)	
1 to 6 times a week	47 (15.0%)	58 (10.4%)	
At least once a day	48 (15.3%)	64 (11.4%)	
**Diet (n, %)**			
Low-fat diet	9 (2.9%)	49 (8.8%)	< 0.001
Normal diet	183 (58.3%)	367 (65.7%)	
High-fat diet	122 (38.8%)	143 (25.6%)	
Regular exercise (n, %)	85 (27.1%)	200 (35.8%)	0.009

AMI, acute myocardial infarction; CAD, coronary artery disease; BMI, body mass index; ACEI, Angiotensin Converting Enzyme Inhibitor; ARB, Angiotensin Receptor Blocker

### Differences in sleep factors between AMI and control groups

Sleep factors were compared between AMI and control groups (Table 2). Significant differences were found in timing of sleep and morning waking, sleep duration at night, frequency of insomnia and night-time waking, sleep medication use, LAN and night work (all *P* < 0.05). However, there were no significant differences in sleep quality, daytime napping and sleep noise.

**Table 2:** Comparisons of sleep factors between AMI and control group

Variables		AMI group (n = 314)	Control group (n = 559)	*P* value
Timing of sleep	Before 22:00	88 (28.0%)	213 (38.1%)	< 0.001
	22:00 to 23:00	68 (21.7%)	174 (31.1%)	
	23:00 to 24:00	58 (18.5%)	118 (21.1%)	
	24:00 and after	100 (31.8%)	54 (9.7%)	
Timing of morning waking	Before 6:00	159 (50.6%)	331 (59.2%)	0.001
	6:00 to 7:00	125 (39.8%)	207 (37.0%)	
	After 7:00	30 (9.6%)	21 (3.8%)	
Sleep duration	< 6 h	78 (24.8%)	62 (11.1%)	< 0.001
	6-9 h	220 (70.1%)	476 (85.1%)	
	> 9 h	16 (5.1%)	21 (3.8%)	
Sleep quality	Very poor	17 (5.4%)	25 (4.5%)	0.105
	Fairly poor	38 (12.1%)	99 (17.7%)	
	Fairly good	126 (40.1%)	230 (41.1%)	
	Very good	133 (42.4%)	205 (36.7%)	
Insomnia frequency	Never	125 (39.8%)	168 (30.1%)	0.037
	≤ 3 times/month	116 (36.9%)	256 (45.8%)	
	4–6 times/month	44 (14.0%)	76 (13.6%)	
	7–12 times/month	12 (3.8%)	29 (5.2%)	
	> 12 times/month	17 (5.5%)	30 (5.3%)	
Sleep medication use	Yes	12 (3.8%)	43 (7.7%)	0.029
LAN	Level 1^a^	9 (2.9%)	9 (1.6%)	< 0.001
	Level 2^b^	28 (8.9%)	37 (6.6%)	
	Level 3^c^	68 (21.7%)	230 (41.2%)	
	Level 4^d^	209 (66.5%)	283 (50.6%)	
Frequency of night-time waking	Never	112 (35.7%)	138 (24.7%)	0.001
	1 time	89 (28.3%)	175 (31.3%)	
	2 times	66 (21.0%)	159 (28.4%)	
	3 times	29 (9.3%)	37 (6.6%)	
	> 3 times	18 (5.7%)	50 (9.0%)	
Night work	Yes	37 (11.8%)	32 (5.7%)	0.002
Daytime napping	Yes	110 (35.0%)	234 (41.9%)	0.051
Sleep noise	Never	292 (93.0%)	530 (94.8%)	0.337
	≤ 3 times/month	4 (1.3%)	9 (1.6%)	
	4–12 times/month	3 (1.0%)	6 (1.1%)	
	> 12 times/month	15 (4.7%)	14 (2.5%)	

LAN, light at night; AMI, acute myocardial infarction

^a^They could read comfortably; ^b^ They could barely read; ^c^ They could see only the hazy outline of the bedroom; ^d^ Participants wore a mask to keep out light or they could not see their hand in front of their face

### Associations between sleep factors and AMI

The result of Spearman correlation between sleep factors in 873 patients is reported in Additional file 4. There were no correlations between timing of sleep and sleep medication use, daytime napping and sleep noise. Therefore, when we analyzed the association between the timing of sleep and AMI, the three sleep factors without correlation with the timing of sleep were adjusted in multivariate logistic regression. The same method was applied to analyze the other sleep factors.

Table 3 presents the results of logistic regression analyses. The timing of sleep and morning waking, sleep duration, sleep medication use, LAN, frequency of night-time waking, night work and daytime napping were significantly associated with the risk of AMI in crude analyses (all *P* < 0.05). After mutually adjustment for other sleep factors and demographic characteristics, the timing of sleep (24:00 and after), the timing of morning waking (after 7:00) and sleep duration (< 6 h) were related with increased risk of AMI (OR = 4.005, *P* < 0.001, OR = 2.544, *P* = 0.011 and OR = 2.968, *P* < 0.001, respectively). Sleep medication use and LAN exposure (level 3) were associated with lowered risk of AMI (OR = 0.441, *P* = 0.029 and OR = 0.243, *P* = 0.009).

**Table 3:** Crude and adjusted odds ratios (ORs) and 95% confidence intervals (CIs) of sleep factors for AMI

Variables		Crude OR (95% CI)	*P* value	Adjusted OR (95% CI)^e^	*P* value	Adjusted OR (95% CI)^f^	*P* value
Timing of sleep	Before 22:00	1		1		1	
	22:00 to 23:00	0.946 (0.650, 1.376)	0.771	0.935 (0.625, 1.400)	0.745	0.937 (0.624, 1.407)	0.755
	23:00 to 24:00	1.190 (0.797, 1.776)	0.395	1.124 (0.719, 1.757)	0.607	1.149 (0.732, 1.803)	0.546
	24:00 and after	4.482 (2.963, 6.780)	< 0.001	3.866 (2.352, 6.354)	< 0.001	4.005 (2.418, 6.634)	< 0.001
Timing of morning waking	Before 6:00	1		1		1	
	6:00 to 7:00	1.257 (0.939, 1.683)	0.124	1.076 (0.773, 1.499)	0.765	1.194 (0.826, 1.725)	0.347
	After 7:00	2.974 (1.650, 5.359)	< 0.001	1.960 (1.020, 3.767)	0.043	2.544 (1.239, 5.225)	0.011
Sleep duration	6-9 h	1		1		1	
	< 6 h	2.722 (1.881, 3.940)	< 0.001	2.629 (1.770, 3.905)	< 0.001	2.968 (1.951, 4.516)	< 0.001
	> 9 h	1.648 (0.844, 3.221)	0.144	1.857 (0.913, 3.777)	0.088	1.696 (0.795, 3.616)	0.172
Sleep quality	Very poor	1		1		1	
	Fairly poor	0.564 (0.275, 1.161)	0.120	0.599 (0.277, 1.295)	0.193	0.631 (0.288, 1.381)	0.249
	Fairly good	0.806 (0.419, 1.548)	0.517	0.805 (0.399, 1.626)	0.546	0.888 (0.434, 1.818)	0.745
	Very good	0.954 (0.496, 1.834)	0.888	0.905 (0.447, 1.830)	0.781	1.018 (0.495, 2.093)	0.962
Insomnia frequency	> 12 times/month	1		1		1	
	Never	1.313 (0.693, 2.486)	0.403	1.103 (0.557, 2.186)	0.779	1.361 (0.676, 2.742)	0.388
	≤ 3 times/month	0.800 (0.424, 1.508)	0.490	0.738 (0.376, 1.449)	0.377	0.908 (0.455, 1.811)	0.784
	4–6 times/month	1.022 (0.507, 2.060)	0.952	1.082 (0.515, 2.274)	0.836	1.212 (0.568, 2.589)	0.619
	7–12 times/month	0.730 (0.297, 1.793)	0.493	1.059 (0.409, 2.740)	0.906	1.224 (0.461, 3.252)	0.684
Sleep medication use	No	1		1		1	
	Yes	0.477 (0.248, 0.918)	0.027	0.577 (0.289, 1.152)	0.119	0.441 (0.212, 0.918)	0.029
LAN	Level 1^a^	1		1		1	
	Level 2^b^	0.757 (0.266, 2.154)	0.602	0.661 (0.220, 1.990)	0.462	0.466 (0.147, 1.477)	0.194
	Level 3^c^	0.296 (0.113, 0.774)	0.013	0.282 (0.102, 0.778)	0.015	0.243 (0.084, 0.700)	0.009
	Level 4^d^	0.739 (0.288, 1.893)	0.528	0.695 (0.257, 1.875)	0.472	0.598 (0.212, 1.684)	0.330
Frequency of night-time waking	Never	1		1		1	
	1 time	0.627 (0.439, 0.895)	0.010	0.825 (0.559, 1.220)	0.335	0.878 (0.589, 1.309)	0.525
	2 times	0.511 (0.350, 0.748)	0.001	0.751 (0.488, 1.156)	0.194	0.766 (0.494, 1.189)	0.235
	3 times	0.966 (0.559, 1.668)	0.900	1.745 (0.940, 3.240)	0.078	1.851 (0.984, 3.484)	0.056
	> 3 times	0.444 (0.245, 0.803)	0.007	0.687 (0.357, 1.319)	0.259	0.566 (0.288, 1.113)	0.099
Night work	No	1		1		1	
	Yes	2.200 (1.341, 3.609)	0.002	1.407 (0.819, 2.417)	0.216	0.648 (0.299, 1.404)	0.272
Daytime napping	No	1		1		1	
	Yes	0.749 (0.562, 0.997)	0.048	0.765 (0.564, 1.037)	0.084	0.765 (0.554, 1.056)	0.104
Sleep noise	Never	1		1		1	
	≤ 3 times/month	0.807 (0.246, 2.642)	0.723	0.628 (0.176, 2.248)	0.475	0.715 (0.190, 2.683)	0.619
	4–12 times/month	0.908 (0.225, 3.655)	0.891	0.792 (0.185, 3.385)	0.753	0.914 (0.204, 4.104)	0.906
	> 12 times/month	1.945 (0.926, 4.085)	0.079	1.836 (0.833, 4.044)	0.132	1.926 (0.813, 4.563)	0.136

AMI, acute myocardial infarction; LAN, light at night; ORs, odds ratios; CIs, confidence Intervals

^a^They could read comfortably; ^b^ They could barely read; ^c^ They could see only the hazy outline of the bedroom; ^d^ Participants wore a mask to keep out light or they could not see their hand in front of their face; ^e^ Adjusted for age, sex, BMI, residence, education level, history of diabetes, hypertension and dyslipidemia, history of coronary artery disease, use of statins, antiplatelet agents, β-blockers, ACEI or ARB, smoking, drinking, diet and regular exercise; ^f^Adjusted for age, sex, BMI, residence, education level, history of diabetes, hypertension and dyslipidemia, history of coronary artery disease, use of statins, antiplatelet agents, β-blockers, ACEI or ARB, smoking, drinking, diet and regular exercise, and adjusted mutually for other sleep factors without significant relationships with each other

### Subgroup analysis of the associations between sleep factors and AMI

The associations between sleep factors and AMI changed in stratified analysis by age (Table 4). In subjects with age ≤ 65 years, the timing of morning waking (after 7:00) and daytime napping were related with increased and reduced risk of AMI respectively (OR = 3.006, *P* = 0.007 and OR = 0.645, *P* = 0.046). In those age > 65 years, the frequency of night-time waking (3 times) was associated with increased risk of AMI (OR = 3.467, *P* = 0.035). Significant associations were observed between late sleep timing (24:00 and after) and short sleep duration (< 6 h) and AMI in both age groups (*P* < 0.001).

**Table 4:** Subgroup analysis of the association between sleep factors and AMI according to age

Variables		≤ 65 years (n = 554)		> 65 years (n = 319)	
		Adjusted OR (95% CI)^e^	*P* value	Adjusted OR (95% CI)^e^	*P* value
Timing of sleep	Before 22:00	1		1	
	22:00 to 23:00	0.814 (0.466,1.422)	0.469	0.884 (0.440,1.775)	0.729
	23:00 to 24:00	0.800 (0.437,1.465)	0.469	1.970 (0.897,4.324)	0.091
	24:00 and after	3.328 (1.773,6.245)	< 0.001	16.827 (4.492,63.026)	< 0.001
Timing of morning waking	Before 6:00	1		1	
	6:00 to 7:00	1.136 (0.728,1.772)	0.574	1.114 (0.568,2.184)	0.754
	After 7:00	3.006 (1.357,6.663)	0.007	1.460 (0.224,9.493)	0.692
Sleep duration	6-9 h	1		1	
	< 6 h	2.522 (1.506,4.225)	< 0.001	5.121 (2.222,11.807)	< 0.001
	> 9 h	1.607 (0.417,6.189)	0.490	2.449 (0.912,6.574)	0.076
Sleep quality	Very poor	1		1	
	Fairly poor	0.841 (0.290,2.441)	0.750	0.657 (0.165,2.614)	0.551
	Fairly good	2.067 (0.789,5.411)	0.139	0.447 (0.122,1.638)	0.224
	Very good	1.939 (0.738,5.091)	0.179	0.645 (0.175,2.381)	0.510
Insomnia frequency	> 12 times/month	1		1	
	Never	2.875 (1.001,8.260)	0.050	0.915 (0.299,2.801)	0.877
	≤ 3 times/month	2.107 (0.737,6.019)	0.164	0.419 (0.140,1.255)	0.120
	4–6 times/month	1.577 (0.515,4.829)	0.425	1.493 (0.447,4.990)	0.515
	7–12 times/month	1.642 (0.343,7.867)	0.535	0.908 (0.220,3.744)	0.894
Sleep medication use	No	1		1	
	Yes	0.361 (0.132,0.988)	0.047	0.480 (0.136,1.695)	0.254
LAN	Level 1^a^	1		1	
	Level 2^b^	0.408 (0.096,1.744)	0.227	0.257 (0.016,4.162)	0.339
	Level 3^c^	0.272 (0.070,1.049)	0.059	0.061 (0.004,0.860)	0.038
	Level 4^d^	0.536 (0.144,2.004)	0.354	0.324 (0.025,4.269)	0.391
Frequency of night-time waking	Never	1		1	
	1 time	0.814 (0.511,1.296)	0.385	1.465 (0.572,3.749)	0.426
	2 times	0.521 (0.299,0.906)	0.021	1.580 (0.636,3.928)	0.325
	3 times	2.097 (0.855,5.139)	0.106	3.467 (1.091,11.019)	0.035
	> 3 times	0.724 (0.273,1.919)	0.516	0.537 (0.156,1.857)	0.326
Night work	No	1		1	
	Yes	1.057 (0.589,1.894)	0.853	–	0.999
Daytime napping	No	1		1	
	Yes	0.645 (0.419,0.992)	0.046	1.041 (0.561,1.930)	0.899
Sleep noise	Never	1		1	
	≤ 3 times/month	0.633 (0.160,2.511)	0.515	0.960 (0.043,21.574)	0.980
	4–12 times/month	0.645 (0.106,3.913)	0.633	1.468 (0.253,8.521)	0.668
	> 12 times/month	2.146 (0.737,6.250)	0.161	–	–

AMI, acute myocardial infarction; LAN, light at night; OR, odds ratio; CI, confidence interval

^a^They could read comfortably; ^b^ They could barely read; ^c^ They could see only the hazy outline of the bedroom; ^d^ Participants wore a mask to keep out light or they could not see their hand in front of their face; ^e^ Adjusted for sex, BMI, residence, education level, history of diabetes, hypertension and dyslipidemia, history of coronary artery disease, use of statins, antiplatelet agents, β-blockers, ACEI or ARB, smoking, drinking, diet and regular exercise, and adjusted mutually for other sleep factors without significant relationships with each other

### Associations between sleep factors and Gensini score

The median value of Gensini score was 42.0, and the high and low score groups included 352 and 357 patients respectively. The characteristics comparisons between high and low score groups are shown in Table 5. Statistically significant correlations were found between Gensini score and the timing of sleep, sleep duration, sleep quality, sleep medication use and the frequency of night-time waking (all *P* < 0.05). However, after adjusting for demographic characteristics in Table 5, the associations between the timing of sleep, sleep medication use and the frequency of night-time waking and Gensini score disappeared. Only the association between short sleep duration and Gensini score remained significant after adjustment for both demographic characteristics and other sleep factors without relationships with each other (OR = 2.374, *P* < 0.001) (Table 6).

**Table 5:** Demographic characteristics of patients in high and low Gensini score groups

Variables	High score group (> 42.0) (n = 352)	Low score group (≤ 42.0) (n = 357)	*P* value
Age (years)	63 (53, 69)	62 (54, 69)	0.786
Men (n, %)	276 (78.4%)	250 (70.0%)	0.013
BMI (kg/m^2^)	24.8 (22.8, 27.3)	24.7 (22.8, 26.9)	0.503
Residence in cities (n, %)	268 (76.1%)	248 (69.5%)	0.052
**Education level (n, %)**			
Illiterate	47 (13.4%)	69 (19.3%)	0.012
Elementary school	68 (19.3%)	90 (25.2%)	
Junior high school	112 (31.8%)	89 (24.9%)	
Senior high school and technical secondary school	67 (19.0%)	68 (19.0%)	
Junior college or above	58 (16.5%)	41 (11.5%)	
Diabetes (n, %)	84 (23.9%)	69 (19.3%)	0.145
Hypertension (n, %)	209 (59.4%)	217 (60.8%)	0.759
Dyslipidemia (n, %)	21 (6.0%)	21 (5.9%)	1.000
Family history of CAD (n, %)	16 (4.5%)	13 (3.6%)	0.574
Statins (n, %)	19 (5.4%)	17 (4.8%)	0.735
Antiplatelet agents (n, %)	29 (8.2%)	30 (8.4%)	1.000
β-blockers (n, %)	15 (4.3%)	31 (8.7%)	0.021
ACEI or ARB (n, %)	75 (21.3%)	102 (28.6%)	0.030
**Smoking (n, %)**			
Never	121 (34.4%)	154 (43.1%)	0.044
Former	46 (13.1%)	53 (14.8%)	
< 20 cigarettes/day	53 (15.1%)	44 (12.3%)	
≥ 20 cigarettes/day	132 (37.5%)	106 (29.7%)	
**Drinking (n, %)**			
No	189 (53.7%)	199 (55.7%)	0.048
1 to 3 times a month	77 (21.9%)	54 (15.1%)	
1 to 6 times a week	45 (12.8%)	44 (12.3%)	
At least once a day	41 (11.6%)	60 (16.8%)	
**Diet (n, %)**			
Low-fat diet	20 (5.7%)	16 (4.5%)	0.040
Normal diet	196 (55.7%)	232 (65.0%)	
High-fat diet	136 (38.6%)	109 (30.5%)	
Regular exercise (n, %)	102 (29.0%)	126 (35.3%)	0.077

CAD, coronary artery disease; BMI, body mass index; ACEI, Angiotensin Converting Enzyme Inhibitor; ARB, Angiotensin Receptor Blocker

**Table 6:** Crude and adjusted odds ratios (ORs) and 95% confidence intervals (CIs) of sleep factors for Gensini score

Variables		Crude OR (95% CI)	*P* value	Adjusted OR (95% CI)^e^	*P* value	Adjusted OR (95% CI)^f^	*P* value
Timing of sleep	Before 22:00	1		1		1	
	22:00 to 23:00	1.076 (0.733, 1.580)	0.708	1.036 (0.682, 1.573)	0.868	1.075 (0.705,1.641)	0.737
	23:00 to 24:00	1.140 (0.757, 1.717)	0.531	1.132 (0.714, 1.795)	0.599	1.153 (0.724,1.835)	0.549
	24:00 and after	1.815 (1.183, 2.784)	0.006	1.671 (0.991, 2.821)	0.054	1.683 (0.989,2.866)	0.055
Timing of morning waking	Before 6:00	1		1		1	
	6:00 to 7:00	1.248 (0.914, 1.705)	0.163	1.179 (0.828, 1.680)	0.361	1.324 (0.903,1.940)	0.151
	After 7:00	1.335 (0.724, 2.464)	0.355	0.987 (0.499, 1.952)	0.969	1.250 (0.593,2.634)	0.557
Sleep duration	6-9 h	1		1		1	
	< 6 h	2.070 (1.380, 3.106)	< 0.001	2.221 (1.447, 3.409)	< 0.001	2.374 (1.523,3.703)	< 0.001
	> 9 h	1.502 (0.732, 3.079)	0.267	1.442 (0.674, 3.085)	0.346	1.234 (0.554,2.750)	0.606
Sleep quality	Very poor	1		1		1	
	Fairly poor	0.355 (0.159, 0.795)	0.012	0.386 (0.166, 0.898)	0.027	0.435 (0.183,1.034)	0.060
	Fairly good	0.565 (0.269, 1.184)	0.130	0.583 (0.269, 1.264)	0.171	0.691 (0.310,1.539)	0.365
	Very good	0.553 (0.264, 1.161)	0.117	0.577 (0.265, 1.254)	0.165	0.680 (0.304,1.521)	0.348
Insomnia frequency	> 12 times/month	1		1		1	
	Never	0.892 (0.462, 1.725)	0.735	0.830 (0.410, 1.681)	0.605	0.835 (0.411,1.698)	0.619
	≤ 3 times/month	0.611 (0.318, 1.174)	0.139	0.574 (0.288, 1.145)	0.115	0.574 (0.287,1.150)	0.117
	4–6 times/month	0.800 (0.384, 1.667)	0.551	0.834 (0.387, 1.797)	0.643	0.813 (0.376,1.758)	0.598
	7–12 times/month	0.469 (0.173, 1.273)	0.137	0.616 (0.214, 1.776)	0.370	0.598 (0.207,1.727)	0.342
Sleep medication use	No	1		1		1	
	Yes	0.503 (0.265, 0.956)	0.036	0.586 (0.298, 1.153)	0.122	0.512 (0.255,1.028)	0.060
LAN	Level 1^a^	1		1		1	
	Level 2^b^	1.037 (0.338, 3.180)	0.949	1.193 (0.376, 3.785)	0.765	1.006 (0.294,3.442)	0.992
	Level 3^c^	0.626 (0.225, 1.738)	0.368	0.678 (0.237, 1.940)	0.469	0.649 (0.211,1.992)	0.449
	Level 4^d^	0.817 (0.299, 2.236)	0.694	0.900 (0.319, 2.537)	0.842	0.825 (0.274,2.483)	0.732
Frequency of night-time waking	Never	1		1		1	
	1 time	0.965 (0.655, 1.420)	0.856	1.042 (0.682, 1.591)	0.851	1.069 (0.697,1.639)	0.760
	2 times	0.633 (0.424, 0.944)	0.025	0.674 (0.426, 1.067)	0.092	0.672 (0.423,1.068)	0.092
	3 times	0.599 (0.328, 1.092)	0.095	0.725 (0.369, 1.421)	0.349	0.739 (0.375,1.456)	0.382
	> 3 times	0.699 (0.388, 1.258)	0.233	0.627 (0.323, 1.217)	0.168	0.620 (0.318,1.211)	0.162
Night work	No	1		1		1	
	Yes	1.054 (0.618, 1.796)	0.847	0.879 (0.492, 1.572)	0.664	0.540 (0.250,1.166)	0.117
Daytime napping	No	1		1		1	
	Yes	0.810 (0.600, 1.094)	0.170	0.829 (0.603, 1.139)	0.247	0.810(0.583,1.126)	0.211
Sleep noise	Never	1		1		1	
	≤ 3 times/month	3.156 (0.633, 15.751)	0.705	2.233 (0.431, 11.568)	0.338	2.708(0.491,14.953)	0.253
	4–12 times/month	1.052 (0.261, 4.242)	0.676	1.199 (0.286, 5.035)	0.804	1.190 (0.273,5.184)	0.816
	> 12 times/month	2.104 (0.889, 4.983)	0.091	2.540 (1.026, 6.287)	0.044	2.422 (0.935,6.277)	0.069

LAN, light at night; ORs, odds ratios; CIs, confidence intervals

^a^They could read comfortably; ^b^ They could barely read; ^c^ They could see only the hazy outline of the bedroom; ^d^ Participants wore a mask to keep out light or they could not see their hand in front of their face; ^e^ Adjusted for age, sex, BMI, residence, education level, history of diabetes, hypertension and dyslipidemia, history of coronary artery disease, use of statins, antiplatelet agents, β-blockers, ACEI or ARB, smoking, drinking, diet and regular exercise; ^f^ Adjusted for age, sex, BMI, residence, education level, history of diabetes, hypertension and dyslipidemia, history of coronary artery disease, use of statins, antiplatelet agents, β-blockers, ACEI or ARB, smoking, drinking, diet and regular exercise, and adjusted mutually for other sleep factors without significant relationships with each other

## Discussion

Our study demonstrated the associations between sleep factors and AMI risk and CAD severity. Consistent with previous researches, short sleep duration at night was obviously correlated with AMI and CAD severity in this study. Hormonal disorders, like an increase of ghrelin [26] or cortisol [27], contribute to the development of obesity [26], diabetes [28] and inflammation [29]. Both obesity and diabetes are risk factors for AMI, leading to an increase of AMI. Short or long sleep duration increases the incidence of type 2 diabetes [30], stroke [31], CAD [31] and arterial stiffness [32]. Long sleep duration can also accompany with other cardiovascular risk factors, like tobacco use [33] and sedentary lifestyle [34]. But the association between long sleep duration and AMI was not proven in our research, which may be due to its relatively small sample size.

Gensini score has been widely used to assess the extent of CAD in clinic. Coronary artery calcification (CAC), quantitated by CAC score, is a hallmark in the formation and accumulation of atherosclerotic plaque [35]. CAC score and carotid artery intima-media thickness, can be measured as Gensini score to assess the severity of CAD [36]. It was demonstrated that short duration of sleep was associated with higher carotid artery intima-media thickness [37] and incidence of CAC [38]. In our research, short sleep duration was also correlated with high Gensini score after adjusting for demographic characteristics and other sleep factors.

Sleep duration is determined by the time of falling asleep and waking up. Therefore, sleeping late, leading to insufficient sleep, is harmful for both adults [39] and adolescents [40]. Furthermore, circadian misalignment due to late sleeping may increase the risk of hypertension [39], obesity [41] and type 2 diabetes [42]. Evening and morning chronotypes are usually divided according to the preference to choose which time of the day for daily activities [43]. Compared with morning chronotypes, evening chronotypes are more likely to encounter health problems, such as the prevalence of smoking, unhealthy diets, sedentary behavior [42, 44]. In people with evening chronotypes, the incidence of type 2 diabetes rises by 2.5 folds and arterial hypertension by 1.3 folds, independent of sleep duration [42]. A Cox regression analysis reveals a 1.15-fold increased risk of all-cause mortality in individuals with evening chronotypes, also regardless of sleep duration [39]. Different from the two researches above, the interactive effects between sleep duration and chronotypes on cardiovascular risk factors were examined. As showed by the results, long sleep duration combined with evening preference had the highest prevalence for all five cardiovascular risk factors [44].

We also found that AMI onset time was influenced by chronotype. It has been reported that AMI incidence peaks in the morning among patients with morning chronotypes and in the afternoon among patients with evening chronotypes [17]. A large body of studies have focused on the circadian patterns of AMI onset, but the effects of chronotypes on AMI risk have received little attention. Our results showed that late sleeping was associated with a higher risk of AMI, which was not hard to understand from the two sides. On the one hand, circadian misalignment influences AMI onset through regulating heart rate, blood pressure, epinephrine and cortisol [45], platelet aggregability [46], and vascular resistance [47]. Compared to morning chronotypes, evening chronotypes are associated with higher resting heart rate, blood pression, epinephrine and cortisol, all contributing to the occurrence of AMI [42, 48]. On the other hand, people with evening chronotypes have a higher possibility of tobacco use, physical inactivity, obesity and less fruit and vegetables consumption [44]. Besides, late sleeping may raise triglyceride and low-density lipoprotein and lower high-density lipoprotein, thus increasing the risk of AMI [49].

The elderly usually need light when they get up at night, in order to avoid falling down. Due to the more elderly people in the control group, the level 3 of LAN, rather than level 4, was correlated with reduced risk of AMI. In the group with age ≤ 65 years, daytime napping seemed to be a protective factor. The sleep duration in this population is shorter, compared with the elderly, but daytime napping can offer rich benefits.

Several limitations should be noted in our study. First, compared with our questionnaire survey, some sleep measurement tools, like polysomnography, will be more objective but costly. Second, the sample size of this study is relatively small and the majority of enrolled subjects are men. Therefore, the results may not be suitable for the whole population, especially in women with AMI. Third, although patients with history of sleep apnea were excluded, we cannot eliminate the influence of snoring on our results absolutely. Nevertheless, our study involved many aspects of sleep habits and took into account the relationships between sleep factors. Besides, questionnaire survey is economical and convenient for large sample size study.

## Conclusions

Short sleep duration and late sleep timing are important risk factors for AMI. Daytime napping may protect the population aged ≤ 65 years. Our results are meaningful and helpful for people to cultivate good habits of sleeping, consequently playing a role in preventing cardiovascular disease.

## Supplementary Information

**Additional file 1.** Definitions for medical conditions.

**Additional file 3.** Gensini score.

**Additional file 3.** Demographic characteristics comparisons between AMI and CAD group and non-CAD group..

**Additional file 4.** Relationships among sleep factors in 873 cases.

## Data Availability

The data presented in this study are available on request from the corresponding author.

## Abbreviations

AMI: Acute myocardial infarction
CAD: Coronary artery disease
SFQ: Sleep factors questionnaire
ECG: Electrocardiogram
BMI: Body mass index
LAN: Light at night
ORs: Odds ratios
CIs: Confidence intervals
ACEI: Angiotensin converting enzyme inhibitor
ARB: Angiotensin receptor blocker
