# Supplementary material for: Effects of sleep habits on acute myocardial infarction risk and severity of coronary artery disease in Chinese population
Source: BMC Cardiovasc Disord. 2021 Oct 7;21:481. doi: 10.1186/s12872-021-02251-8 (PMC8499531; doi:10.1186/s12872-021-02251-8)
Supplement: Supplementary file 1 — Additional file 1. Definitions for medical conditions. [file 12872_2021_2251_MOESM1_ESM.docx]

**Supplementary information**

**Additional file 1** Definitions for medical conditions

Mental disorders: a syndrome characterized by clinically significant disturbance in an individual's cognition, emotion regulation, or behavior that reflects a dysfunction in the psychological, biological, or developmental processes underlying mental functioning [19]. Sleep apnea: a sleep disorder characterized by intermittent complete and partial airway collapse, resulting in frequent episodes of apnea and hypopnea [20]. Chronic obstructive pulmonary disease: a common preventable and treatable disease characterized by persistent airflow limitation that is usually progressive and associated with an enhanced chronic inflammatory response in the airways and the lung to noxious particles or gases [21]. Stroke sequelae: a suddenly developed sensory and motor dysfunction resulting from brain abnormalities, or imaging studies revealed evidences of brain infarction or hemorrhage related to the symptoms [22]. Arthritis: any disorder that affects the joints. End-stage kidney failure: estimated glomerular filtration rate (eGFR) <15ml/(min·1.73m^2^) or need for renal replacement therapy.

Siesta: a short nap taken in the early afternoon, often after the midday meal. Sleep quality: the degree of excellence in sleep. Sleep duration at night: the time spent asleep during the night. Timing of sleep and waking up: sleep start and end times. Insomnia: difficulties in initiating or maintaining sleep. Night-time waking frequency: the number of sleep interruptions at night. Sleep medication use: medication was needed to initiate or maintain sleep more than one week in total in the last year. Night work: work performed between 20:00 to 6:00. Daytime napping: a short period of sleep during the day. LAN exposure: the average light intensity before asleep at night. Sleep noise: unwanted sounds that affect sleep.
