# Supplementary material for: Effects of sleep habits on acute myocardial infarction risk and severity of coronary artery disease in Chinese population
Source: BMC Cardiovasc Disord. 2021 Oct 7;21:481. doi: 10.1186/s12872-021-02251-8 (PMC8499531; doi:10.1186/s12872-021-02251-8)
Supplement: Supplementary file 2 — Additional file 3. Gensini score. [file 12872_2021_2251_MOESM2_ESM.docx]

**Additional file 2** Gensini score

According to coronary angiographic results, each lesion was assigned a score according to the percentage of stenosis-1 for 25% stenosis, 2 for 50%, 4 for 75%, 8 for 90%, 16 for 99% and 32 for total occlusion. The coefficient of each major coronary artery and each segment was defined as 5 points for left main coronary lesion, 2.5 points for proximal left anterior descending branch and left circumflex artery, 1.5 points for middle left descending artery lesion, 1 point for first diagonal branch and obtuse marginal branches and right coronary artery, 0.5 points for the second diagonal and posterolateral branch of the left circumflex artery. Gensini score was the sum of all segment scores [23].
