# Supplementary material for: Effects of sleep habits on acute myocardial infarction risk and severity of coronary artery disease in Chinese population
Source: BMC Cardiovasc Disord. 2021 Oct 7;21:481. doi: 10.1186/s12872-021-02251-8 (PMC8499531; doi:10.1186/s12872-021-02251-8)
Supplement: Supplementary file 3 — Additional file 3. Demographic characteristics comparisons between AMI and CAD group and non-CAD group. [file 12872_2021_2251_MOESM3_ESM.docx]

**Additional file 3**

**Supplemental Table 1** Demographic characteristics comparisons between AMI and CAD group and non-CAD group

| Variables | AMI group  （n=314） | Control group (n=559) | | *P*  value ^a^ | *P*  value ^b^ |
| --- | --- | --- | --- | --- | --- |
|  |  | CAD group(n=395) | Non-CAD group(n=164) |  |  |
| Age (years) | 59 (51, 68) | 64 (55, 70) | 60 (53, 66) | 0.001 | 0.898 |
| Men (n, %) | 250 (79.6%) | 276 (69.9%) | 85 (51.8%) | 0.003 | <0.001 |
| BMI (kg/m^2^) | 25.1 (22.8, 27.0) | 24.6(22.9,27.1) | 24.4(22.4,26.9) | 0.793 | 0.250 |
| Residence in cities (n, %) | 239 (76.1%) | 277 (70.1%) | 116 (70.7%) | 0.089 | 0.226 |
| **Education level (n, %)** |  |  |  |  |  |
| Illiterate | 40 (12.7%) | 76 (19.3%) | 40 (24.4%) | 0.007 | 0.006 |
| Elementary school | 58 (18.4%) | 100 (25.3%) | 38 (23.2%) |  |  |
| Junior high school | 101 (32.2%) | 100 (25.3%) | 40 (24.4%) |  |  |
| Senior high school and technical secondary school | 63 (20.1%) | 72 (18.2%) | 26 (15.9%) |  |  |
| Junior college or above | 52 (16.6%) | 47 (11.9%) | 20 (12.2%) |  |  |
| Diabetes (n, %) | 54 (17.2%) | 99 (25.1%) | 20 (12.2%) | 0.013 | 0.183 |
| Hypertension (n, %) | 165 (52.5%) | 261 (66.1%) | 79 (48.2%) | <0.001 | 0.387 |
| Dyslipidemia (n, %) | 20 (6.4%) | 22 (5.6%) | 6 (3.7%) | 0.749 | 0.289 |
| Family history of CAD  (n, %) | 15 (4.8%) | 14 (3.5%) | 2 (1.2%) | 0.449 | 0.066 |
| Statins (n, %) | 11 (3.5%) | 25 (6.3%) | 8 (4.9%) | 0.120 | 0.468 |
| Antiplatelet agents (n, %) | 21 (6.7%) | 38 (9.6%) | 13 (7.9%) | 0.173 | 0.708 |
| β-blockers (n, %) | 13 (4.1%) | 33 (8.4%) | 9 (5.5%) | 0.031 | 0.499 |
| ACEI or ARB (n, %) | 54 (17.2%) | 123 (31.1%) | 31 (18.9%) | <0.001 | 0.706 |
| **Smoking (n, %)** |  |  |  |  |  |
| Never | 102 (32.5%) | 173 (43.8%) | 103 (62.8%) | <0.001 | <0.001 |
| Former | 30 (9.6%) | 69 (17.5%) | 13 (7.9%) |  |  |
| <20 cigarettes/day | 46 (14.6%) | 51 (12.9%) | 15 (9.1%) |  |  |
| ≥20 cigarettes/day | 136 (43.3%) | 102 (25.8%) | 33 (20.1%) |  |  |
| **Drinking (n, %)** |  |  |  |  |  |
| No | 154 (49.0%) | 234 (59.3%) | 115 (70.1%) | 0.049 | <0.001 |
| 1 to 3 times a month | 65 (20.7%) | 66 (16.7%) | 22 (13.4%) |  |  |
| 1 to 6 times a week | 47 (15.0%) | 42 (10.6%) | 16 (9.8%) |  |  |
| At least once a day | 48 (15.3%) | 53 (13.4%) | 11 (6.7%) |  |  |
| **Diet (n, %)** |  |  |  |  |  |
| Low-fat diet | 9 (2.9%) | 27 (6.8%) | 22 (13.4%) | 0.012 | <0.001 |
| Normal diet | 183 (58.3%) | 245 (62.0%) | 122 (74.4%) |  |  |
| High-fat diet | 122 (38.8%) | 123 (31.2%) | 20 (12.2%) |  |  |
| Regular exercise (n, %) | 85 (27.1%) | 143 (36.2%) | 57 (34.8%) | 0.010 | 0.092 |

AMI, acute myocardial infarction; CAD, coronary artery disease; BMI, body mass index; ACEI, Angiotensin Converting Enzyme Inhibitor; ARB, Angiotensin Receptor Blocker.

^a^ AMI compared with CAD group; **^b^** AMI compared with non-CAD group.
