# Supplementary material for: Effects of sleep habits on acute myocardial infarction risk and severity of coronary artery disease in Chinese population
Source: BMC Cardiovasc Disord. 2021 Oct 7;21:481. doi: 10.1186/s12872-021-02251-8 (PMC8499531; doi:10.1186/s12872-021-02251-8)
Supplement: Supplementary file 4 — Additional file 4. Relationships among sleep factors in 873 cases. [file 12872_2021_2251_MOESM4_ESM.docx]

**Additional file 4**

**Supplemental Table 2** Relationships among sleep factors in 873 cases

| **r** | Timing of sleep | Timing of morning waking | Sleep duration | Sleep quality | Insomnia frequency | Sleep medication use | LAN | Frequency of night-time waking | Night work | Daytime napping | Sleep noise |
| --- | --- | --- | --- | --- | --- | --- | --- | --- | --- | --- | --- |
| Timing of sleep | — | 0.455^*^ | 0.235^*^ | -0.188^*^ | 0.083^*^ | -0.038 | 0.076^*^ | -0.297^*^ | -0.143^*^ | -0.009 | -0.005 |
| Timing of morning waking | 0.455^*^ | — | -0.002 | 0.049 | -0.062 | 0.032 | 0.060 | -0.220^*^ | -0.118^*^ | -0.036 | -0.012 |
| Sleep duration | 0.235^*^ | -0.002 | — | -0.236^*^ | 0.222^*^ | -0.109^*^ | -0.031 | 0.009 | -0.111^*^ | -0.068^*^ | 0.073^*^ |
| Sleep quality | -0.188^*^ | 0.049 | -0.236^*^ | — | -0.736^*^ | 0.340^*^ | 0.085^*^ | -0.221^*^ | 0.004 | 0.080^*^ | -0.059 |
| Insomnia frequency | 0.083^*^ | -0.062 | 0.222^*^ | -0.736^*^ | — | -0.319^*^ | -0.026 | 0.259^*^ | 0.086^*^ | -0.082^*^ | 0.024 |
| Sleep medication use | -0.038 | 0.032 | -0.109^*^ | 0.340^*^ | -0.319^*^ | — | 0.013 | -0.099^*^ | -0.024 | 0.016 | 0.003 |
| LAN | 0.076^*^ | 0.060 | -0.031 | 0.085^*^ | -0.026 | 0.013 | — | -0.110^*^ | -0.007 | 0.005 | -0.280^*^ |
| Frequency of night-time waking | -0.297^*^ | -0.220^*^ | 0.009 | -0.221^*^ | 0.259^*^ | -0.099^*^ | -0.110^*^ | — | 0.166^*^ | 0.011 | -0.031 |
| Night work | -0.143^*^ | -0.118^*^ | -0.111^*^ | 0.004 | 0.086^*^ | -0.024 | -0.007 | 0.166^*^ | — | 0.080^*^ | -0.056 |
| Daytime napping | -0.009 | -0.036 | -0.068^*^ | 0.080^*^ | -0.082^*^ | 0.016 | 0.005 | 0.011 | 0.080^*^ | — | -0.012 |
| Sleep noise | -0.005 | -0.012 | 0.073^*^ | -0.059 | 0.024 | 0.003 | -0.280^*^ | -0.031 | -0.056 | -0.012 | — |

LAN, light at night; ^*^*P* < 0.05.
